# Critical Assessment of Cell Wall Integrity Factors Contributing to *in vivo* Echinocandin Tolerance and Resistance in *Candida glabrata*

**DOI:** 10.3389/fmicb.2021.702779

**Published:** 2021-06-30

**Authors:** Rocio Garcia-Rubio, Rosa Y. Hernandez, Alissa Clear, Kelley R. Healey, Erika Shor, David S. Perlin

**Affiliations:** ^1^Center for Discovery and Innovation, Hackensack Meridian Health, Nutley, NJ, United States; ^2^Department of Biology, William Paterson University, Wayne, NJ, United States; ^3^Department of Medical Sciences, Hackensack Meridian School of Medicine, Nutley, NJ, United States; ^4^Lombardi Comprehensive Cancer Center, Georgetown University, Washington, DC, United States

**Keywords:** fungal infections, *Candida glabrata*, echinocandins, glucan synthase, cell wall integrity, *YPS1*, *YPK2*, *SLT2*

## Abstract

Fungal infections are on the rise, and emergence of drug-resistant *Candida* strains refractory to treatment is particularly alarming. Resistance to azole class antifungals, which have been extensively used worldwide for several decades, is so high in several prevalent fungal pathogens, that another drug class, the echinocandins, is now recommended as a first line antifungal treatment. However, resistance to echinocandins is also prominent, particularly in certain species, such as *Candida glabrata*. The echinocandins target 1,3-β-glucan synthase (GS), the enzyme responsible for producing 1,3-β-glucans, a major component of the fungal cell wall. Although echinocandins are considered fungicidal, *C. glabrata* exhibits echinocandin tolerance both *in vitro* and *in vivo*, where a subset of the cells survives and facilitates the emergence of echinocandin-resistant mutants, which are responsible for clinical failure. Despite this critical role of echinocandin tolerance, its mechanisms are still not well understood. Additionally, most studies of tolerance are conducted *in vitro* and are thus not able to recapitulate the fungal-host interaction. In this study, we focused on the role of cell wall integrity factors in echinocandin tolerance in *C. glabrata.* We identified three genes involved in the maintenance of cell wall integrity – *YPS1*, *YPK2*, and *SLT2* – that promote echinocandin tolerance both *in vitro* and in a mouse model of gastrointestinal (GI) colonization. In particular, we show that mice colonized with strains carrying deletions of these genes were more effectively sterilized by daily caspofungin treatment relative to mice colonized with the wild-type parental strain. Furthermore, consistent with a role of tolerant cells serving as a reservoir for generating resistant mutations, a reduction in tolerance was associated with a reduction in the emergence of resistant strains. Finally, reduced susceptibility in these strains was due both to the well described *FKS*-dependent mechanisms and as yet unknown, *FKS*-independent mechanisms. Together, these results shed light on the importance of cell wall integrity maintenance in echinocandin tolerance and emergence of resistance and lay the foundation for future studies of the factors described herein.

## Introduction

Invasive fungal infections, in particular candidiasis and candidemia, pose a serious threat to immunocompromised individuals and are associated with high mortality rates (Pfaller and Diekema, 2010). Treatment outcomes critically depend on timely and appropriate use of antifungal therapy. However, therapeutic choices for fungal infections are scarce due to a small number of available drug classes and a rise in antifungal drug resistance (Arastehfar et al., 2020). For several decades, *Candida albicans* has been the predominant infecting *Candida* species worldwide and azole drugs were the primary therapy to prevent or treat *Candida* infections. However, extensive use of azoles has led to an epidemiological shift toward non-*albicans Candida* species with higher azole tolerance or resistance, such as *Candida glabrata* (Enoch et al., 2017). Therefore, another drug class, the echinocandins, is currently recommended as first line therapy, especially for suspected non-*albicans* infections (Perlin, 2011). Alarmingly, however, *C. glabrata* also shows elevated potential relative to other *Candida* species to rapidly develop resistance to echinocandins, resulting in difficult-to-treat multidrug-resistant infections (Pham et al., 2014).

Echinocandins target the fungal-specific enzyme 1,3-β-D-glucan synthase (GS), which is responsible for the biosynthesis of a major cell wall polymer and is essential for building and maintaining the fungal cell wall (Perlin, 2015a). Echinocandin resistance is caused by mutations in GS-encoding *FKS* genes, and these mutations are often associated with therapeutic failure (Shields et al., 2012; Alexander et al., 2014). Although echinocandins are considered fungicidal drugs in *Candida* spp., including *C. glabrata*, we have shown that whereas the vast majority of *C. glabrata* cells do die upon echinocandin exposure *in vitro*, a small subset of cells survives and shows drug tolerance over a wide range of echinocandin concentrations (Healey and Perlin, 2018). In this context, it is important to note that because echinocandins are cidal in *Candida*, whereas azoles are static, we use the word “tolerance” not to indicate growth above minimum inhibitory concentrations (MICs), as has been defined for azoles (Berman and Krysan, 2020), but to refer to the ability of cells to survive a given echinocandin concentration that is expected to kill cells, usually at or above the MIC. Under this definition, strains with increased tolerance show improved survival, whereas strains with decreased tolerance show reduced survival, and this can be observed at one or more concentrations of the drug. Additionally, because MIC assays measure not survival but growth, changes in echinocandin tolerance may or may not correspond to changes in echinocandin MICs. *In vivo*, for example in animal models of infection, tolerance is likewise manifested by an incomplete reduction in fungal burdens upon treatment, i.e., in failure to achieve sterilization (Howard et al., 2011). These tolerant cells likely serve as the cellular reservoir in which resistant mutations arise (Shor and Perlin, 2015); however, despite this key role of drug tolerance in development of drug resistance, the factors underlying echinocandin tolerance in *C. glabrata* are very poorly understood. Furthermore, most studies looking at drug responses of *C. glabrata* have been conducted *in vitro*, providing no information on how drug tolerance is affected *in vivo*.

The fungal cell wall is the target of echinocandin action, and its structure and stability are key factors regulating resistance and tolerance to these drugs. For instance, fungal cells respond to echinocandin exposure by compensatory increases in cell wall chitin content (Walker et al., 2013), and cells with constitutively increased chitin levels have elevated echinocandin resistance (Lee et al., 2012). Conversely, inhibition of chitin biosynthesis by nikkomycin Z, a chitin synthase inhibitor, sensitizes *C. glabrata* cells to caspofungin treatment (Healey et al., 2017). Because the cell wall is also a key interface with the environment, such as the host, fungal cells contain several signaling pathways responsible for cell wall maintenance, which detect cell wall damage and activate the cellular processes necessary for cell wall stabilization and repair (Dichtl et al., 2016). However, the importance of these pathways in echinocandin tolerance has not yet been examined.

In this study, we show that four genes that function in cell wall integrity maintenance (*SLG1*, *YPS1*, *YPK2*, and *SLT2*) contribute to echinocandin tolerance in *C. glabrata in vitro*. Furthermore, we used our murine model of gastrointestinal (GI) colonization to demonstrate that deletion mutants of *YPS1*, *YPK2*, and *SLT2* showed increased GI decolonization by caspofungin, reduced incidence of fungal burden rebound, and reduced emergence of *fks* mutations. Our results identify these cell wall integrity pathways as important contributors to echinocandin tolerance in *C. glabrata* both *in vitro* and *in vivo* and highlight them as potential targets for adjuvant antifungal therapies.

## Materials and Methods

### Ethics Statement

Mice were housed in the Research Animal Facility Biosafety Level-2 at the Center for Discovery and Innovation, Hackensack Meridian Health (Nutley, NJ, United States). The animal facility follows the Public Health Service and National Institute of Health Policy of Humane Care and Use of Laboratory Animals guide. All experimental protocols were approved by the Center for Discovery and Innovation Institutional Animal Care and Use Committee (IACUC).

### Yeast Strains and Media

The *C. glabrata* strains used in this study were ATCC2001HTL, ATCC90030, and the described deletion mutants. Deletion mutants in the ATCC2001HTL background were provided by Dr. Kuchler (Schwarzmüller et al., 2014), whereas deletion mutants in ATCC90030 background were generated in our lab. Cells were cultured in the standard yeast extract-peptone-dextrose (YPD) medium at 37°C, which is the optimal growth temperature for this species.

### Construction of *Candida glabrata* Deletion Mutants

Deletion mutants were generated in-house using a CRISPR-Cas9 targeted integration replacing the desired ORF by a nourseothricin (NAT)-resistance cassette. The deletion construct containing the NAT-resistance cassette flanked by regions homologous to the locus of interest was amplified from genomic DNA of the deletion mutants in ATCC2001HTL background. All primers used are listed in Supplementary Table 1. Integration of the deletion cassettes was performed using CRISPR as described previously (Shor et al., 2019). Briefly, cells were made competent for electroporation using the Frozen-EZ yeast transformation kit (Zymo Research) according to the manufacturer’s instructions and then electroporated with Cas9-gRNA complex and the DNA containing the deletion construct. Transformants were selected on NAT-containing plates and validated by PCR amplification and sequencing of the targeted locus using external primers (Supplementary Table 1). At least two independent transformants were generated for every deletion mutant. All primers were ordered from Integrated DNA Technologies (Coralville, IA, United States), and all Sanger sequencing of the above-described constructs was done by Genewiz (South Plainfield, NJ, United States).

### *In vitro* Tolerance Assay

Fresh 1 ml YPD cultures of 10^7^ *C. glabrata* cells were incubated at 37°C with shaking (150 rpm) for 24 h in the presence of a range of micafungin or caspofungin concentrations (0, 0.015, 0.06, 0.25, 1, and 4 μg/ml). After 24 h, 0.1 ml of the appropriate dilutions for each culture was plated onto YPD plates. Colony forming units (CFU) were determined and survival percentage was obtained by normalizing the CFU obtained from cultures treated with the indicated concentration of drug to non-treated controls. At least three biological replicates were performed for every strain and condition.

### Echinocandin Susceptibility Testing

Micafungin (Astellas, Deerfield, IL, United States) and caspofungin (Merck, Rahway, NJ, United States) susceptibility testing was performed using a broth microdilution method following CLSI standards (Clinical and Laboratory Standards Institute (CLSI), 2017) with some modifications. The media used was YPD broth and the final concentrations tested ranged from 0.0035 to 2 μg/ml in two-fold increasing concentrations. MICs were visually read after 24 h of incubation at 37°C and at least three biological replicates were performed.

### Murine Model of *Candida glabrata* GI Colonization

The GI model of *C. glabrata* colonization was performed as described (Healey et al., 2017) with some modifications. 6-week-old female CF-1 immunocompetent mice (Charles River Laboratories) were treated subcutaneously, daily from day-2 to 15, with 320 mg/kg of piperacillin-tazobactam (PTZ, 8:1 ratio, AuroMedics Pharma LLC, East Windsor, NJ, United States) to clear native intestinal bacterial microbiota. On day 0, mice were inoculated via oral gavage with approximately 1.5⋅10^8^ CFU of *C. glabrata* in 0.1 ml of PBS. In this manner mice were colonized with ATCC2001HTL, ATCC90030 or their derivative mutants. Daily administration of 20 mg/kg of caspofungin (Merck, Rahway, NJ, United States) or PBS intraperitoneal was initiated on day 3 post inoculation and continued through day 15. Fresh fecal samples were collected every other day throughout the experiment to assess fungal burden in the GI tract.

The experiment comparing ATCC2001HTL and ATCC90030 strains, contained six mice in the caspofungin treated group. Additionally, two mice were in the colonization control group (treated with PBS alone) for each strain. In the experiment studying the effects of cell wall integrity mutants, the caspofungin treated groups contained five mice per deletion mutant; since two independent mutants were analyzed per deletion, ten mice were analyzed in total per one gene deletion. Also for every deletion, one mouse was included as an untreated control (treated with PBS alone). All mice in the PBS alone control groups maintained a constant fungal burden of 10^6^–10^8^ CFU/g of stool.

### Determination of *in vivo* Caspofungin Resistance Development

Caspofungin-resistant colonies were identified through selection of fecal samples on YPD plates supplemented with caspofungin (2 μg/ml), PTZ (16 μg/ml), and chloramphenicol (20 μg/ml), followed by PCR amplification and sequencing of *FKS1* and *FKS2*. Since clinical echinocandin resistance in *C. glabrata* is nearly always associated to amino acid substitutions in the hotspot regions of *FKS1* and *FKS2* (Perlin, 2015b), we sequenced a region of 0.8–1 kb containing each hotspot to ensure detection of all mutations associated with a change in susceptibility (Supplementary Table 1).

### Non-*Candida glabrata* Species Identification

When species specific *FKS1/2* primers were not successful at PCR amplification, we suspected colonization by a non-*C. glabrata* species. In this case, we performed a species determination analysis using primers ITS1 and ITS4 (White et al., 1990). One mouse of the untreated control in group *yps1Δ* was excluded from the study as colonization was established by a non-*C. glabrata* species, *Cyberlindnera fabianii*, confirmed by ITS amplification and sequencing (Supplementary Table 1).

### Chitin Content Determination

The assessment of cell wall chitin content was performed as described (Costa-de-Oliveira et al., 2013) with some modifications. As the authors recommended, 2.5 μg/ml of calcofluor white (CFW) was used to stain chitin, the mean intensity of fluorescence was detected from three independent experiments using BV-421 filter followed by flow cytometry analysis using FlowJo^TM^ software v10.6.1 (BD Biosciences). Chitin percentage of fluorescence was calculated relative to the parental strain ATCC90030. To confirm that CFW was staining chitin, strains ATCC2001HTL and its chitin synthase deletion mutant (ATCC2001HTL *chs3Δ*) were included as controls.

### Statistical Analysis

Data analyses were performed using GraphPad Prism 8 software. Unpaired *t*-test was used to determine statistically significant differences for the *in vitro* tolerance assay, for fungal burdens of colonization between experimental groups (wild-type vs. deletion mutants) and for the differences in chitin content. To establish if the differences in rebound frequency were significant between experimental groups, the Freeman-Halton extension of the Fisher exact probability test was used. In all cases, *p*-values < 0.05 (two-tailed) were considered statistically significant.

## Results

### Identification of Four Candidate Genes Whose Deletions Reduce Echinocandin Tolerance

**FIGURE 1:**
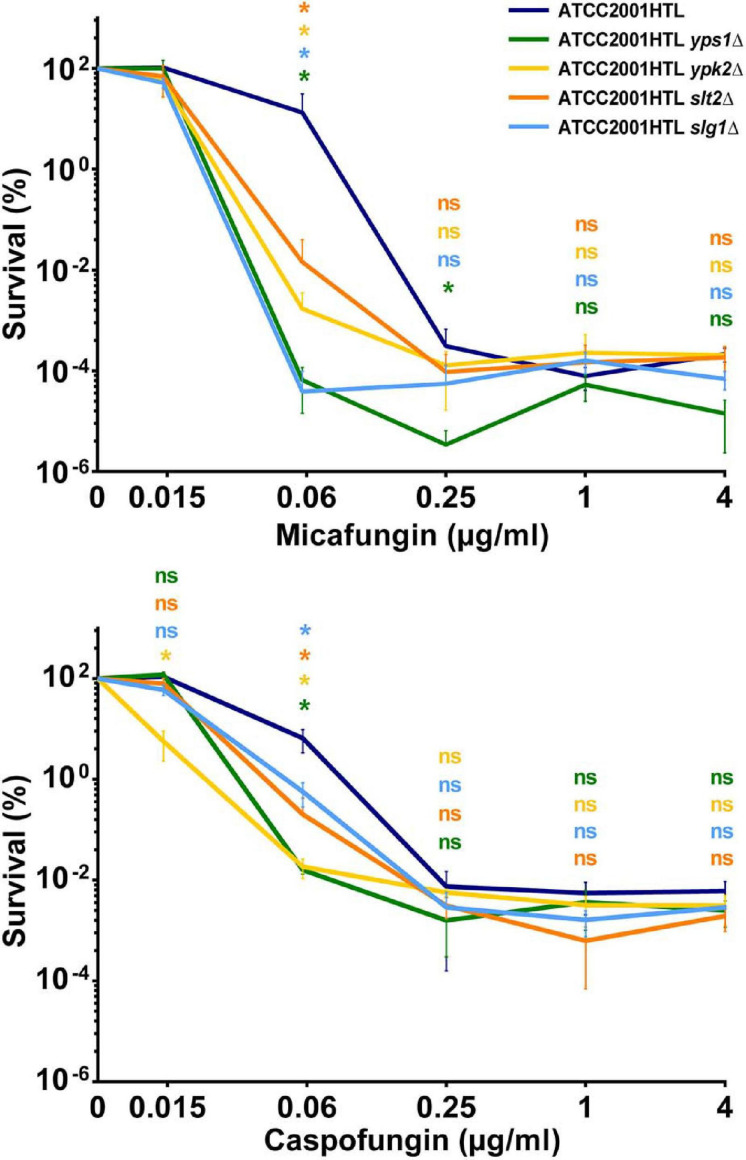
Deletions of genes involved in cell wall maintenance exhibited significantly reduced tolerance to micafungin and caspofungin. Survival percentage was obtained by normalizing the colony forming units (CFU) obtained from cultures treated with the indicated drug concentrations normalized to non-treated controls for each strain. A decrease in drug tolerance was observed in one or more drug concentrations of micafungin or caspofungin in *yps1Δ, ypk2Δ*, *slt2Δ*, and *slg1Δ* mutants after 24 h-drug exposure to a wide range of concentrations. Results were calculated from at least three independent biological replicates. The statistical significance was calculated using an unpaired *t*-test and is indicated with an asterisk (*) or with n.s. (not significant).

To identify new genes that contribute to *C. glabrata* echinocandin tolerance, we examined a collection of fifteen *C. glabrata* deletion mutants (Table 1) generated as part of a systematic deletion collection in the ATCC2001HTL background (Schwarzmüller et al., 2014). These mutants were chosen because they were sensitive to cell wall damaging agents (some to caspofungin) (Schwarzmüller et al., 2014; Rosenwald et al., 2016) and/or because these genes or their orthologs in *Saccharomyces cerevisiae* were known to function in cell wall maintenance (Table 1). We measured the survival of each knock-out strain after 24-h exposure to micafungin over a wide range of concentrations. As discussed previously (Healey et al., 2017), such survival assays are a better indicator of drug tolerance than the standard MIC assay, which measures growth but not survival and therefore cannot detect non-growing surviving cells that possess the potential for resistance development. Whereas most of the deletion mutants showed no or minor changes in micafungin tolerance (Supplementary Figure 1), four of them – *yps1Δ*, *ypk2Δ*, *slt2Δ*, and *slg1Δ* – resulted in significantly reduced tolerance to micafungin and caspofungin at one or more concentrations of the drug (Figure 1) and were therefore selected for further studies.

**TABLE 1:** Fifteen *Candida glabrata* genes whose deletion mutants were analyzed for echinocandin tolerance *in vitro*.

Cg gene name	Cg systematic name	Sc ortholog	Gene ontology category**	Function
*CBK1*	CAGL0J06072g	*CBK1*	Cell wall organization	Orthologs have protein serine/threonine kinase activity
*CHS5*	CAGL0G00814g	*CHS5*	Vacuole, Golgi	Orthologs have small GTPase binding activity
*CKA1*	CAGL0I05192g	*CKA1*	Signal transduction	Orthologs have protein serine/threonine kinase activity
*CRZ1*	CAGL0M06831g	*CRZ1*	Transcriptional regulation	Transcription factor and downstream component of the calcineurin signaling pathway
*GAS2*	CAGL0M13849g	*GAS1*	Cell wall organization	Putative glycoside hydrolase of the Gas/Phr family
*MID2*	CAGL0G00858g	*MID2*	Signal transduction	Orthologs have transmembrane signaling receptor activity
*MOT3*	CAGL0K03003g	*MOT3*	Transcriptional regulation	Orthologs have DNA-binding transcription factor activity, and RNA polymerase II activating transcription factor binding
*RLM1*	CAGL0H05621g	*RLM1*	Transcriptional regulation	Putative transcription factor with a predicted role in cell wall integrity
*RTA1*	CAGL0K00715g	*RTA1*	Other	Putative protein involved in 7-aminocholesterol resistance
*SBE2*	CAGL0A02486g	*SBE2*	Cell wall organization	Orthologs have role in fungal-type cell wall organization
SLG1*	CAGL0F01507g	*SLG1*	Signal transduction	Putative sensor of stress-activated signaling and ortholog involved in maintenance of cell wall integrity
SLT2*	CAGL0J00539g	*SLT2*	Signal transduction	Mitogen-activated protein kinase with a role in cell wall integrity
*SNF1*	CAGL0M08910g	*SNF1*	Transcriptional regulation	Putative serine/threonine protein kinase required for trehalose utilization
YPK2*	CAGL0K03399g	*YPK2*	Signal transduction	Orthologs have protein serine/threonine kinase activity
YPS1*	CAGL0M04191g	*YPS1*	Cell wall organization	Yapsin family aspartic protease

*Genes selected in this work for further analysis *in vitro* and *in vivo*.

**Based on *Saccharomyces* Genome Database (https://yeastgenome.org/) gene ontology annotations.

### Selection of a *Candida glabrata* Strain for *in vivo* Studies of Echinocandin Tolerance

**FIGURE 2:**
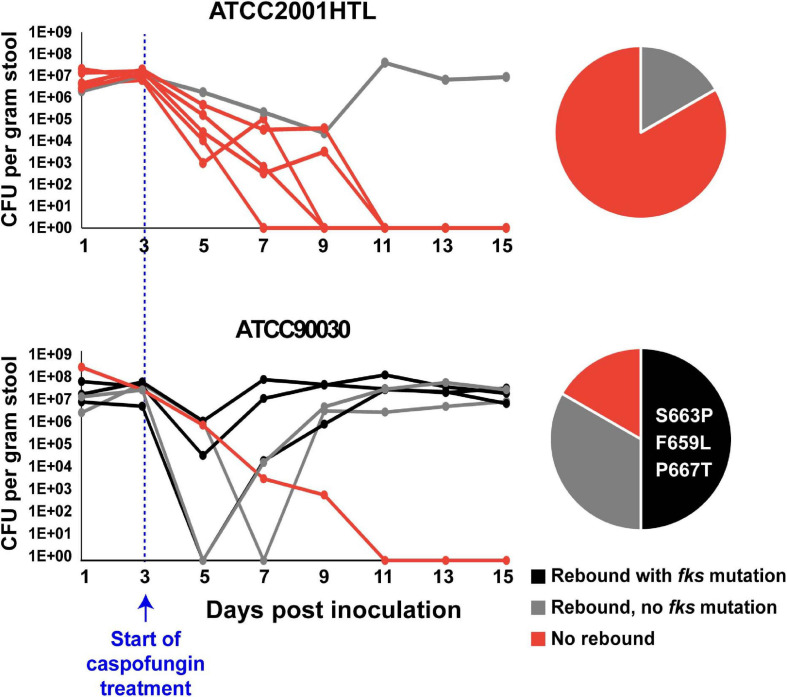
*Candida glabrata* strain ATCC90030 was more tolerant to caspofungin than strain ATCC2001HTL in the mouse model of gastrointestinal (GI) colonization, leading to a higher rate of rebound and emergence of echinocandin resistant *fks*-mutants. Six mice per group were colonized with the indicated *C. glabrata* strains and exposed to daily high-dose caspofungin treatment starting at day 3 post-colonization. CFU per gram of stool were plotted for each day of sample collection, and frequencies for rebound and *fks* mutations are indicated in pie charts. Zero and three *fks*-mutants were obtained in ATCC2001HTL and ATCC90030 background, respectively. All obtained mutations were in the *FKS2* gene, and the specific amino acid changes are listed in the pie chart.

We next wished to investigate the contribution of these genes to echinocandin tolerance *in vivo* using a mouse model of *C. glabrata* GI colonization previously developed in our laboratory (Healey et al., 2017). This model recapitulates important aspects of *C. glabrata* commensalism and emergence of drug resistance because the GI tract is a primary source of *Candida* colonization and has also been described as a reservoir of antimicrobial resistant microorganisms (Donskey, 2004). Briefly, in this model the mouse GI tract is sterilized using antibiotics and colonized via oral gavage with *C. glabrata*. After 3 days of colonization, stably colonized mice are exposed to high-dose daily caspofungin (20 mg/kg body weight). *In vivo*, tolerance is manifested as an initial decline in GI fungal burdens but not true sterilization, whereas resistance, or a reduction in drug susceptibility, is manifested as a rebound of GI burdens to near their initial levels. Our expectation was that strains with reduced echinocandin tolerance *in vitro* would also show reduced tolerance *in vivo*, resulting in improved rates of sterilization by the drug and decreased rebound of the fungal burden after treatment. However, we found that we could not test this hypothesis in ATCC2001HTL background, because this strain showed a very low rate of rebound in the GI model, making it impossible to detect a further decrease potentially caused by a mutant (Figure 2). Thus, we performed a pilot GI colonization experiment with a different strain, ATCC90030, which showed a higher level of tolerance and rebound after caspofungin treatment than ATCC2001HTL (Figure 2). As expected, the rebound in fungal burden was accompanied by the emergence of reduced susceptibility to caspofungin in the yeast isolated from mouse fecal pellets, and it was often (though not always) associated with *fks* mutations. Three different *fks* mutations were identified, all in the *FKS2* gene (S663P, F659L, and P667T) and previously shown to have clinical relevance. Thus, ATCC90030 was chosen as the strain background, in which to test the contribution of *YPS1*, *YPK2*, *SLT2*, and *SLG1* to echinocandin tolerance *in vivo*.

### Deletions of *YPS1*, *YPK2*, and *SLT2* in ATCC90030 Background Increase the Susceptibility to Echinocandin *in vitro*

**FIGURE 3:**
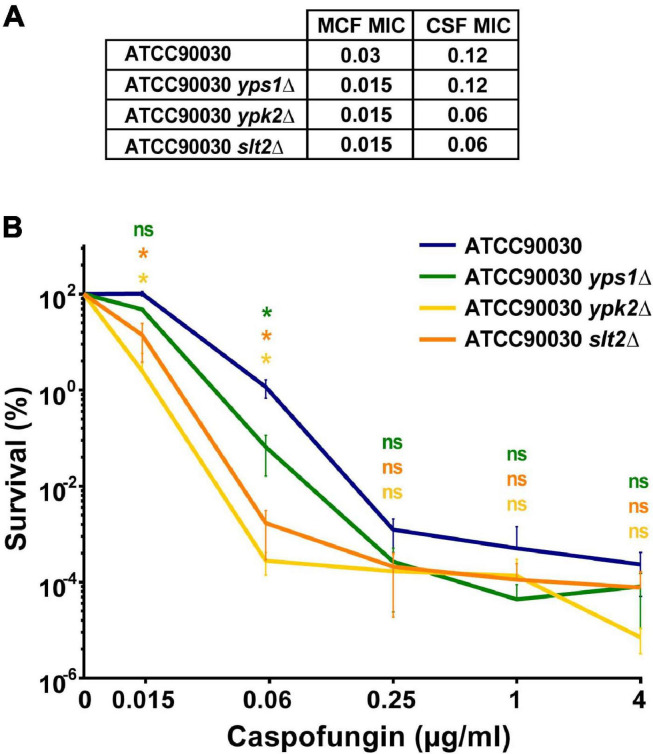
*In vitro* echinocandin hyper-susceptibility of *yps1Δ, ypk2Δ*, and *slt2Δ* was confirmed in the ATCC90030 strain background. **(A)** Minimum inhibitory concentration (MIC) susceptibility testing to caspofungin and micafungin showed that the deletion mutants had lower or identical MIC-values than the wild-type parental strain. Echinocandin concentrations are shown in μg/ml. **(B)** The *in vitro* tolerance assay confirmed that the mutants exhibited reduced drug tolerance compared to the parental ATCC90030 strain. Statistical significance was calculated using an unpaired *t*-test; *p*-value ≤ 0.05 is indicated with an asterisk (*), *p*-value > 0.05 is indicated as n.s. (not significant).

A CRISPR-based approach was used to delete the four genes, separately, in the *C. glabrata* ATCC90030 strain and replace them with *NAT* resistance marker. Despite several attempts, we were unable to knock out *SLG1*. Interestingly, a recent saturation transposon mutagenesis study, conducted *in C. glabrata* BG2 strain background, indicated that *SLG1* confers a fitness defect in *C. glabrata* (Gale et al., 2020), which may explain our inability to obtain this mutant. If this is the case, the original *slg1Δ* mutant generated in the ATCC2001HTL background may have a suppressor mutation, which improves fitness. Alternatively, ATCC90030 may be more sensitive to loss of *SLG1* than ATCC2001HTL, e.g., due to transcriptional rewiring of the cell wall integrity pathway. For the other three genes, two independent knock-out mutants were generated and analyzed in subsequent assays. In all cases the two mutants showed identical phenotypes, so we present their combined data. Using susceptibility testing, we found that all three mutants resulted in a two- to four-fold reduction in caspofungin and micafungin MIC compared to the ATCC90030 parent strain (Figure 3A). We also performed an *in vitro* echinocandin tolerance assay using caspofungin to align with our GI colonization model. After 24-h caspofungin exposure, all three mutants showed reduced survival relative to the parent strain (Figure 3B), recapitulating the echinocandin tolerance defect observed in the ATCC2001HTL background (Figure 1) and confirming the roles of these genes in caspofungin tolerance *in vitro*.

### Deletions of *YPS1*, *YPK2*, and *SLT2* Led to Improved Treatment Efficacy, Decreased Emergence of Fungal Burden and Reduced Caspofungin Susceptibility

**FIGURE 4:**
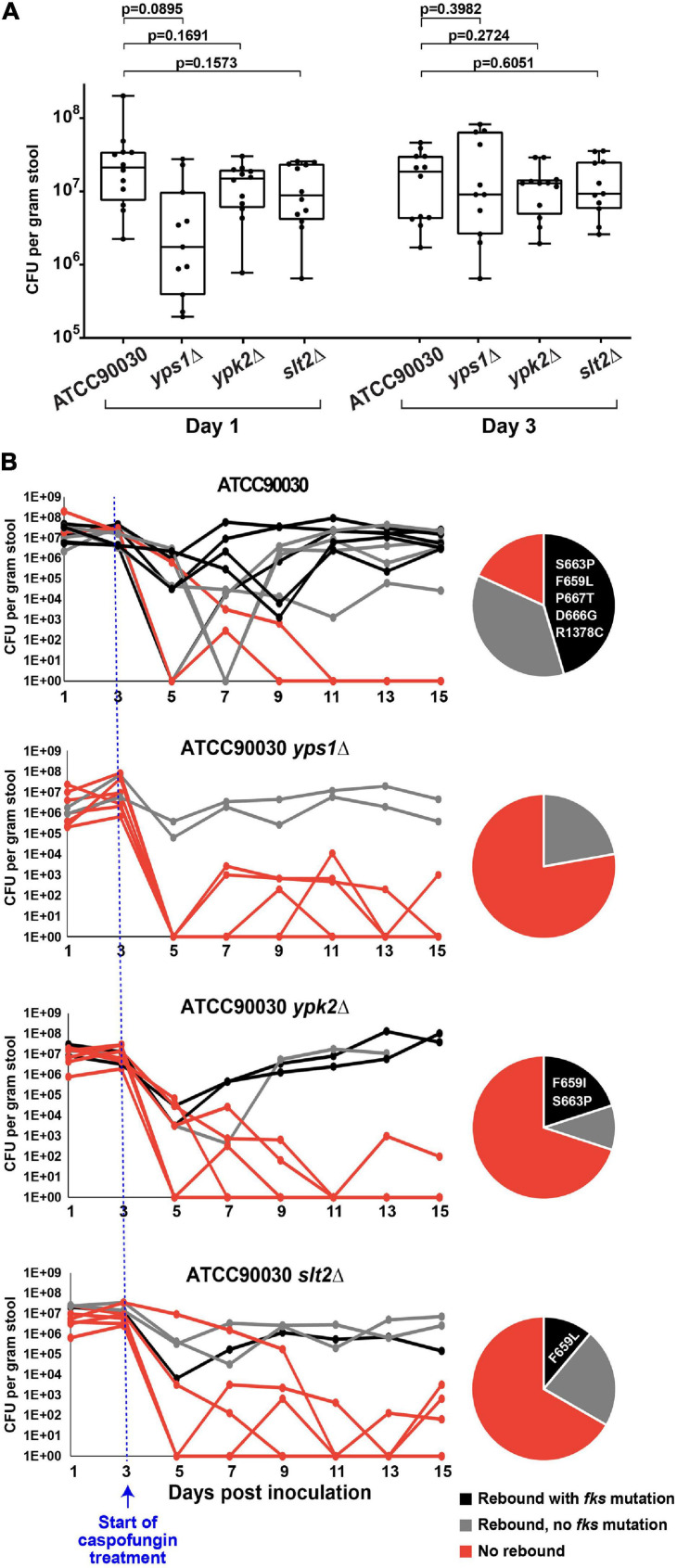
Three cell wall integrity mutants (*yps1Δ*, *ypk2Δ*, and *slt2Δ*) exhibited reduced caspofungin tolerance and decreased emergence of strains with reduced susceptibility to caspofungin in the mouse model of GI colonization. **(A)** Prior to the start of caspofungin treatment, all mice reached and maintained a fungal burden of 10^6^–10^8^ CFU/g of stool, with non-significant differences between groups. **(B)** Caspofungin treatment was more effective at sterilizing the GI of mice colonized with the cell wall integrity mutants. Mice colonized with the ATCC90030 parental strain showed a higher rebound of fungal burden relative to the deletion mutants, which was due to strains both with and without *fks* mutations. All obtained mutations were in the *FKS2* gene, and the specific amino acid changes are listed in the pie charts.

To determine whether the three mutants under examination also alter echinocandin susceptibility *in vivo*, we used our *C. glabrata* GI colonization model (Healey et al., 2017). As described above, the mouse GI tract was sterilized using antibiotics and colonized via oral gavage with strain ATCC90030 or its knock-out derivatives. Stable colonization of the GI tract was established by all strains by day 3 (Figure 4A), at which point the mice were started on high-dose daily caspofungin (20 mg/kg). After 2 days of caspofungin treatment there was a drop in GI fungal burdens in all treated mice independent of genotype. However, at later time points, some mice showed a rebound in fungal burdens due to strains with reduced susceptibility, and this occurred at different rates in mice colonized with the wild-type parent strain compared to the mice colonized with the knock-out mutants (Figure 4B). For instance, for the ATCC90030 wild-type strain, there was a rebound of the fungal burden in nine out of 11 mice (82%), reaching burdens of 10^5^–10^8^ CFU/g of stool by the end of the experiment (15 days post-colonization, 12 days post-treatment) despite daily administration of high-dose caspofungin (Figure 4B). The remaining two mice out of 11 colonized with ATCC90030 eventually showed full sterilization. As expected, rebound was associated with reduced susceptibility to caspofungin in the yeast isolated from fecal pellets, and slightly more than half of the isolated strains contained *fks* mutations (Figure 4B). In contrast, mice inoculated with cell wall integrity deletion mutants showed significantly higher rates of sterilization and reduced levels of rebound of fungal burden (Figure 4B). For instance, for the *yps1Δ* mutant, only two out of nine colonized mice showed rebound after treatment, while the remaining seven showed near or full sterilization (Figure 4B, *p* = 0.02 vs. wild-type strain), and no *fks* mutations were detected in the two mice with fungal rebound. Similarly, for the *ypk2Δ* and *slt2Δ* mutants, three mice showed rebound, while seven and six, respectively, showed near or full sterilization (Figure 4B, *p* = 0.02 for *ypk2Δ* and *p* = 0.03 for *slt2Δ*). As was the case with the wild-type strain, about half of the yeast strains isolated from mice with fungal rebound contained *fks* mutations (Figure 4B). Thus, all three cell wall integrity mutants led to reduced echinocandin tolerance, decreased emergence of strains with reduced susceptibility to caspofungin, and improved treatment efficacy *in vivo*.

### Fungal Rebound After Treatment Is Associated With Elevated Caspofungin MICs Due to Both *fks*-Dependent and Independent Mechanisms

**FIGURE 5:**
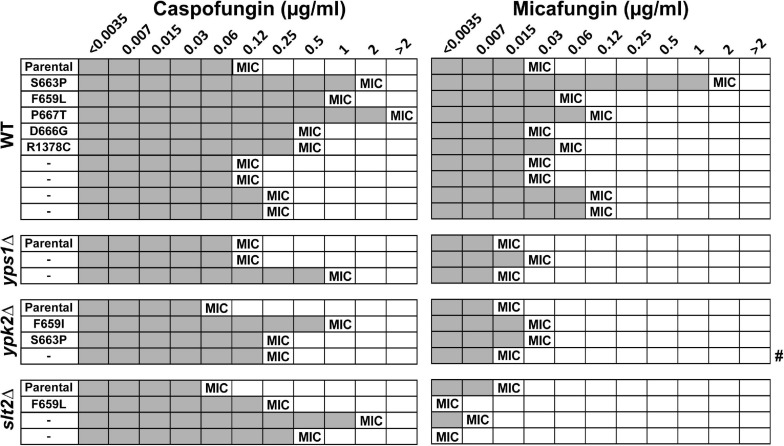
Fungal rebound was predominantly due to strains with elevated MICs to caspofungin but not necessarily micafungin. All strains with *fks* mutations showed reduced susceptibility to caspofungin relative to the parental strain. Gray shading indicates visible growth in drug-containing media, and the MIC is defined as the lowest concentration at which there is at least a 50% decrease in growth. # indicates one mouse in the *ypk2Δ* group, which had to be sacrificed on day 13 and for which day 13 colonies not day 15 were therefore analyzed.

To ask whether fungal rebound was associated with reduced echinocandin susceptibility, we measured the MICs of caspofungin and micafungin in strains isolated from the GI tract of mice with fungal rebound 15 days after colonization (except for one mouse in the *ypk2Δ* group, which had to be sacrificed on day 13 and for which day 13 colonies were therefore analyzed) and compared them to the MICs of parental strains. As expected, the majority of rebound strains, including all strains carrying *fks* mutations, showed elevated caspofungin MICs relative to parental strains (Figure 5). Interestingly, many of the rebound strains, including two *fks* mutants (wild-type D666G and *slt2Δ* F659L) and most *FKS* wild-type strains, did not show elevated micafungin MICs, indicating that the change in susceptibility was specific to the drug used for treatment. Two *fks* mutations were isolated in multiple backgrounds: the *fks2-S663P* mutation was isolated both in the wild-type and the *ypk2Δ* strains, whereas the *fks2-F659L* mutation was isolated both in wild-type and *slt2Δ* strains. Interestingly, in both cases the MIC of the wild-type strain with the mutation was several-fold higher than the MIC of the mutant with the same mutation (Figure 5), suggesting that cell wall maintenance defects reduce echinocandin tolerance even for strains with mutant GS. Finally, this analysis identified a number of strains lacking *fks* mutations but nevertheless showing elevated caspofungin MICs (Figure 5), suggesting that these strains had as yet unknown *FKS*-independent mechanisms leading to reduced drug susceptibility. One mechanism by which *Candida* cells tolerate cell wall stress is by up-regulation of chitin levels (Reinoso-Martín et al., 2003; Cota et al., 2008), so we examined the chitin content of the non-FKS mutants using CFW staining followed by flow cytometry analysis. However, we found no significant differences in CFW content between non-FKS rebound strains and their parental strains (Supplementary Figure 2), indicating that chitin levels may not be the mechanism underlying their elevated echinocandin MICs.

## Discussion

In this study, we assessed the contribution of several *C. glabrata* cell wall integrity genes to echinocandin tolerance *in vitro* and *in vivo* and to emergence of strains with reduced drug susceptibility in *C. glabrata* colonizing the mouse GI tract. We show that deletion of four genes involved in the maintenance of cell wall integrity, *SLG1, YPS1*, *YPK2*, and *SLT2*, increased the susceptibility of *C. glabrata* to two echinocandin class drugs, micafungin and caspofungin. Consistent with that finding, deletion mutants of *YPS1*, *YPK2*, and *SLT2* were more efficiently eliminated from the mouse GI system by high-dose caspofungin treatment, with an associated reduction in the emergence of mutants with decreased susceptibility to caspofungin, including resistant mutants.

Cell wall integrity in both *C. glabrata* and closely related, well studied budding yeast *S. cerevisiae* is controlled protein kinase C (PKC), which upon cell wall damage triggers a Mitogen Activated Protein (MAP) kinase cascade that culminates with the phosphorylation and activation of Slt2p MAP kinase (Reinoso-Martín et al., 2003; Cota et al., 2008). Activated Slt2p phosphorylates and activates multiple downstream target proteins implicated in the expression of cell wall-related genes, including GS subunit *FKS2* (Jung and Levin, 1999; de Nobel et al., 2000). Thus, the mechanistic role of *SLT2* in cell wall integrity has been extensively studied in *S. cerevisiae*, and its importance has also been examined in *C. glabrata*. For instance, the *C. glabrata slt2Δ* strain was shown to have decreased tolerance to several cell wall damaging agents, such as congo red, CFW, and micafungin (Miyazaki et al., 2010). Furthermore, overexpression of *SLT2* has been linked to an increase in chitin content and incomplete *in vitro* killing by caspofungin in *C. glabrata* and in *S. cerevisiae* (Reinoso-Martín et al., 2003; Cota et al., 2008). Thus, the reduced caspofungin and micafungin MIC of the *slt2Δ* mutants is consistent with a decreased expression of one or both *FKS* genes and/or other cell wall components controlled by this pathway. Interestingly, our data also show a genetic interaction between *slt2Δ* and *fks2-F659L* mutation, wherein this mutation raised the micafungin MIC of the wild-type strain but reduced the micafungin MIC of the *slt2Δ* mutant. This difference suggests that the effect of this mutation on the drug-target interaction may be dependent on the expression level of the target (GS), which is regulated by *SLT2*. Overall, the results presented here reinforce the importance of *SLT2* in echinocandin tolerance in *C. glabrata* and provide the first evidence for a role of this gene in echinocandin tolerance in *C. glabrata* colonizing a mammalian host and in emergence of drug resistance *in vivo*.

Like *SLT2*, *YPK2* encodes a protein kinase; however, significantly less information is available about Ypk2p function. Ypk2p and its closely related ortholog Ypk1p have been studied in *S. cerevisiae*, where deletion of *YPK1* results in slow growth, whereas *ypk2Δ* shows no apparent phenotypic defects (Chen et al., 1993). However, *S. cerevisiae* cells deleted for both genes are inviable, indicating that these genes have functionally redundant roles in cell viability. Interestingly, the cells carrying inactivating mutations in both *YPK1* and *YPK2* lyse rapidly unless placed in osmotically supportive medium (Roelants et al., 2002). Furthermore, the inviability of the *ypk1Δ ypk2Δ* mutant is rescued by overexpression of *EXG1*, a major exo-1,3-beta-glucanase of the cell wall, supporting a role of these genes in cell wall integrity. Indeed, genetic analyses have placed *YPK1* and *YPK2* in a cell wall integrity pathway that acts in parallel with the *PKC1*-dependent pathway (Roelants et al., 2002). Our results suggest that in *C. glabrata* at least Ypk2p is also necessary for the maintenance of cell wall integrity, contributing to echinocandin tolerance both *in vitro* and in the animal host.

Yps1p is a member of the yapsin protein family, which are GPI-linked aspartyl proteases with established roles in cell wall integrity and in virulence (Krysan et al., 2005). In *S. cerevisiae* the yapsin family contains five members, whereas *C. glabrata* has eleven yapsins, showing an expansion of this family in *C. glabrata*. Yapsin functions have been predominantly studied in *S. cerevisiae*, where deletion mutants have defects in cell wall glucan incorporation (but not in glucan synthesis), with mannan and chitin levels increased as a compensatory response (Krysan et al., 2005). The quintuple yapsin mutant is sensitive to cell wall-disrupting agents, showing dramatically altered cell wall composition and temperature-induced lysis. Phenotypic analysis of individual yapsin deletion mutants in *S. cerevisiae* highlighted the important role of ScYps1p in cell wall maintenance: ScYps1 was shown to promote glucan incorporation and/or retention within the cell wall in response to cell wall stress and remodeling, acting as part of the transcriptional response to cell wall stress mediated by the Pkc1 signaling cascade (Heinisch et al., 1999). In agreement with these data, Sc*yps1Δ* mutant is sensitive to caspofungin, congo red, and caffeine, and its overexpression during cell wall stress helps maintain cell wall glucan homeostasis (Krysan et al., 2005). *C. glabrata* yapsins are less well studied, but CgYps1p seems to have a role in cell wall regulation in the context of maintaining pH homeostasis, helping reduce total beta-glucan levels in the cell wall in acidic environments (Bairwa and Kaur, 2011). *In vivo* models have shown that yapsins are essential for *C. glabrata* virulence in a murine model of systemic infection (Kaur et al., 2007) as well as responsible for epithelial cell damage in an experimental oral infection model with *C. albicans* (Albrecht et al., 2006). In agreement with the consensus role of yapsins in cell wall integrity maintenance, our study demonstrates that CgYps1 is necessary for echinocandin tolerance both *in vitro* and *in vivo*, and for emergence of echinocandin resistance *in vivo*.

Clinical echinocandin resistance in *C. glabrata* is strictly associated with amino acid substitutions in specific hotspot regions of integral membrane proteins Fks1p and Fks2p (Perlin, 2015b), and most of these mutations directly correlate with clinical failure (Garcia-Effron et al., 2009; Shields et al., 2012; Alexander et al., 2014). All *fks* mutations described in this work occurred in the *FKS2* gene, which is in agreement with the previous observations that echinocandin resistance is more frequently associated with mutation in *FKS2* in *C. glabrata* (Arendrup and Perlin, 2014). Notably, seven isolates out of nine harbored *fks* mutations (F659I, F659L, S663P, D666G, and P667T) that also have been identified among human clinical isolates, and several of these have been associated with clinical failure (Garcia-Effron et al., 2009; Alexander et al., 2014). Modifications of *FKS2* at positions F659 or R1378 have also been previously described (Shields et al., 2012; Bienvenu et al., 2019); however, as far as we know, the F659I and R1378C mutations are novel amino acid changes associated with reduced susceptibility identified for the first time in this work. Finally, the rebound in fungal burden was not always associated with *fks* mutations, indicating additional mechanisms that will be explored in future studies. The clinical significance of this finding is intriguing but unclear. Whereas compensatory increases in cell wall chitin content have been linked to increased echinocandin tolerance (Reinoso-Martín et al., 2003; Krysan et al., 2005; Cota et al., 2008), we found no significant differences in the chitin levels of these strains compared to their parental strain. The elevated MICs of rebound strains could also be linked to changes in plasma membrane lipid composition, similar to what we have demonstrated for *Aspergillus fumigatus*, where two membrane sphingolipids, dihydrosphingosine and phytosphingosine, rendered GS insensitive to echinocandins (Satish et al., 2019). Supporting the sphingolipid hypothesis, whereas all isolates with *fks* mutations showed reduced susceptibility to caspofungin (elevated caspofungin MICs), this was not the case for micafungin. Indeed, several strains lacking *fks* mutations and one strain containing an *fks* mutation (wild-type background, *fks2-D666G*) had higher MIC values to caspofungin than their respective parental strains, but unaltered, or sometimes decreased, MICs to micafungin. This phenomenon was reminiscent of the so-called “CRS-MIS phenotype” observed in a set of *C. glabrata* mutants derived *in vitro* that showed caspofungin reduced susceptibility (CRS) combined with micafungin increased susceptibility (MIS) (Healey et al., 2012). The CRS-MIS phenotype was shown to be connected to changes in membrane sphingolipids modulating the echinocandin-GS interaction. Thus, it is possible that some or all mutants isolated in our study that show a CRS-MIS-like phenotype also have altered membrane sphingolipid composition, resulting in these particular changes in GS sensitivity to echinocandin class drugs.

In summary, the experiments presented here evaluate echinocandin tolerance and emergence of strains than are less susceptible to killing by caspofungin in *C. glabrata* colonizing the animal host, uncovering an important role of multiple cell wall integrity maintenance mechanisms in these processes. These experiments lay the foundation for future work examining other components of these pathways, the mechanistic details underpinning echinocandin tolerance, and identification of new ways to manipulate these mechanisms to enhance the efficacy of drug treatment and prevent emergence of resistance.

## Data Availability Statement

The original contributions presented in the study are included in the article/Supplementary Material, further inquiries can be directed to the corresponding authors.

## Ethics Statement

The animal study was reviewed and approved by Center for Discovery and Innovation Institutional Animal Care and Use Committee (IACUC).

## Author Contributions

RG-R, RH, and AC performed the experiments. RG-R and ES performed the data analysis. RG-R, KH, ES, and DP designed the experiments. RG-R, ES, and DP wrote the manuscript. ES and DP supervised, coordinated, and directed the project. All authors contributed to the article and approved the submitted version.

## Conflict of Interest

DP receives research support and/or serves on advisory boards for Amplyx, Cidara, Scynexis, N8 Medical, Matinas, Merck, Regeneron, and Pfizer. RG-R, RH, AC, KH, and ES declare that the research was conducted in the absence of any commercial or financial relationships that could be construed as a potential conflict of interest.
