# Supplementary material for: Critical Assessment of Cell Wall Integrity Factors Contributing to in vivo Echinocandin Tolerance and Resistance in Candida glabrata
Source: Front Microbiol. 2021 Jun 30;12:702779. doi: 10.3389/fmicb.2021.702779 (PMC8298035; doi:10.3389/fmicb.2021.702779)
Supplement: Supplementary file 1 [file Data_Sheet_1.docx]

Supplementary Material

# Supplementary Figures

**Supplementary Figure 1. Most of the deletion mutants tested showed no or minor changes in micafungin tolerance.** Survival percentage was obtained by normalizing the colony forming units (CFU) obtained from cultures treated for 24 hours with the indicated micafungin concentrations normalized to non-treated controls for each strain. Results were calculated from at least three independent biological replicates.


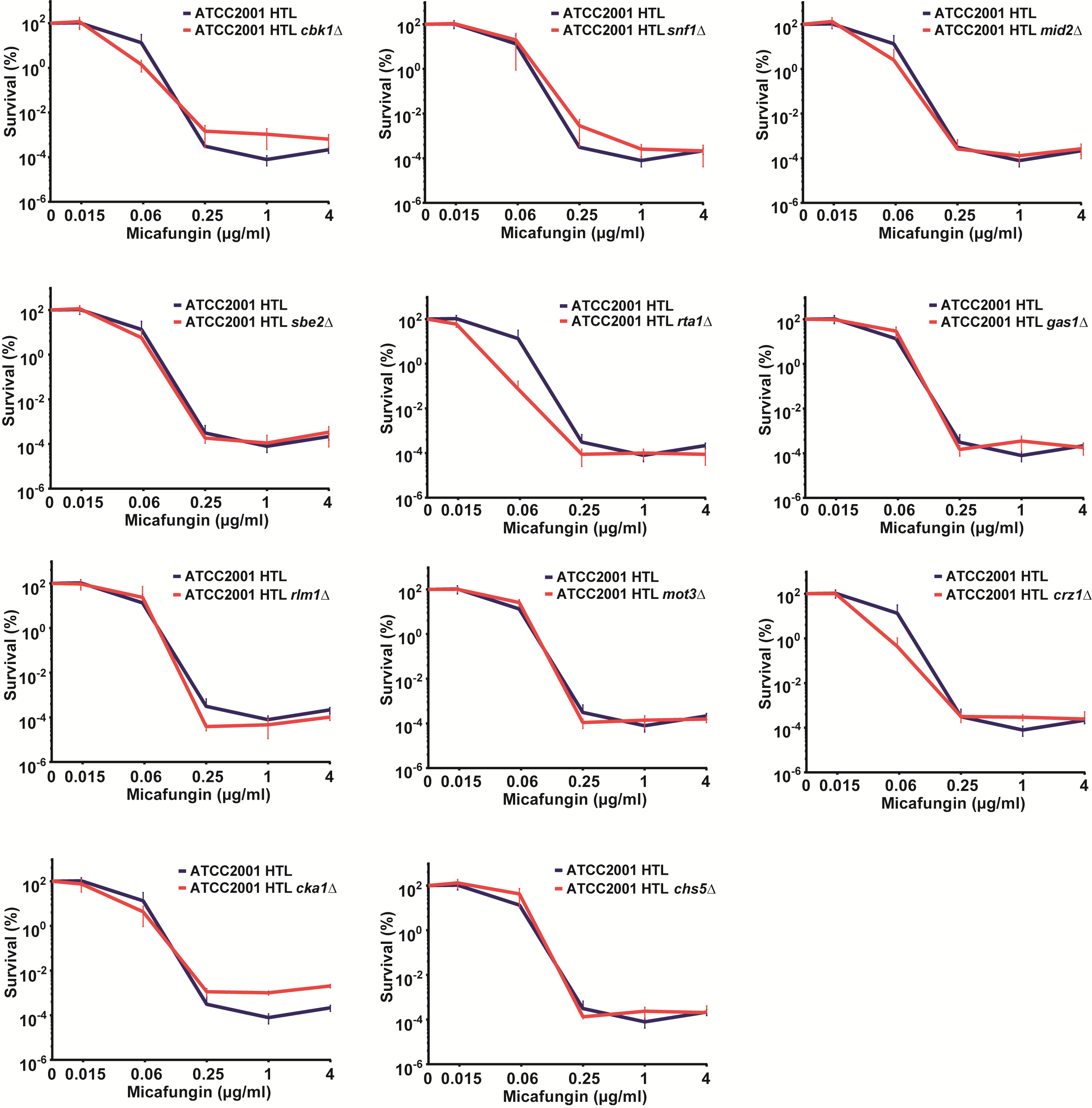


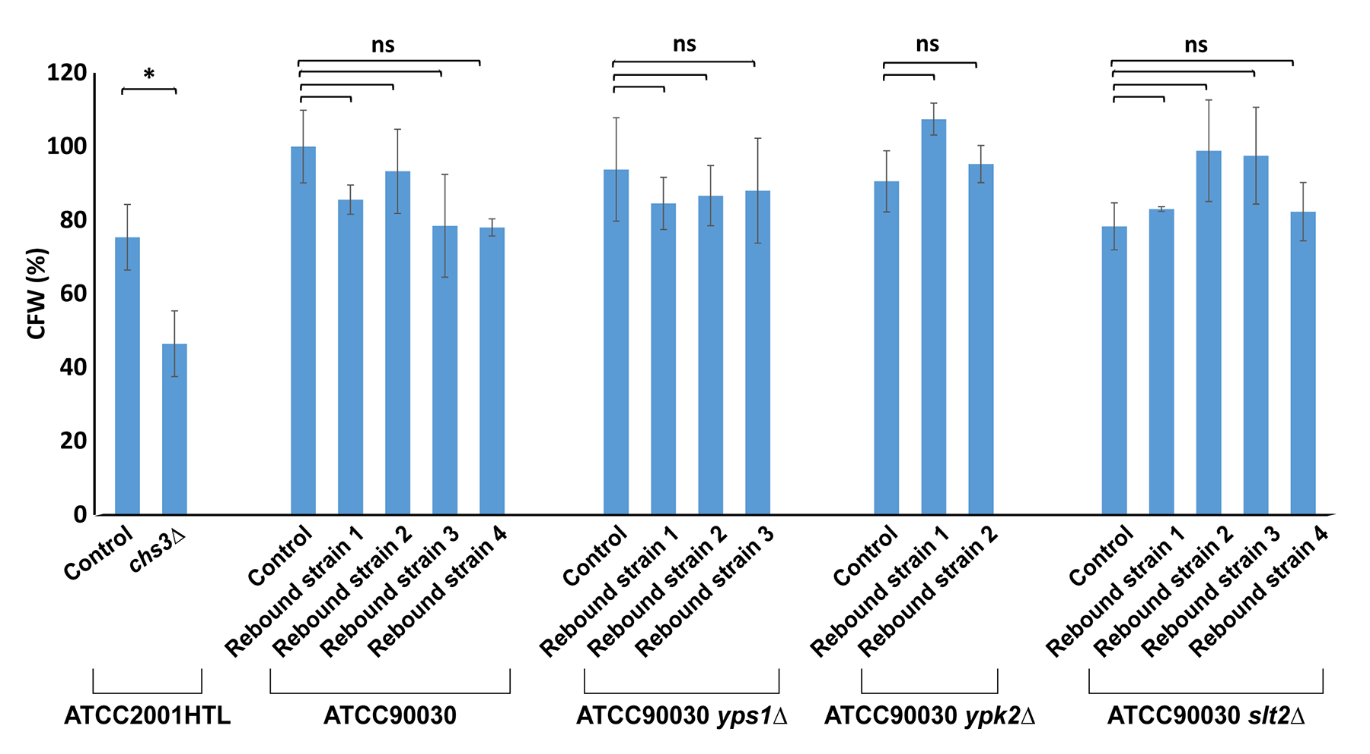


**Supplementary Figure 2. No differences in chitin content were observed in any of the rebound strains.** Chitin determination was performed using calcofluor white (CFW) staining followed by flow cytometry analysis. As a control, we used chitin synthase mutant *chs3∆* in the ATCC2001HTL background, which showed a ~30% reduction in CFW staining relative to its parental wild-type strain. The CFW+ percentage for each strain was obtained by normalizing to the wild-type ATCC90030 strain.

# Supplementary Tables

**Supplementary table 1.** Sequences of primers used in this study. All primers were used for amplification and sequencing

| **Name** | **Description** | **Sequence (5’-3’)** | **Ref** |
| --- | --- | --- | --- |
| ESP193 | yps1 fwd replacement template | CAAGAGAAGCAAAGAAGCAAAGC | This  study |
| ESP194 | yps1 rv replacement template | CCCGATAGCAAAGCTGCGTG |  |
| ESP195 | yps1 fwd NAT validation | GCAAGATGCGAAATTTAGCAGG |  |
| ESP196 | yps1 rv NAT validation | TGCTACCCTGCTGGTCAAGC |  |
| ESP189 | ypk2 fwd replacement template | TCGAAACAAGGGACGCCAACAC |  |
| ESP190 | ypk2 rv replacement template | CGAAAGGTAGCGAGACGGTG |  |
| ESP191 | ypk2 fwd NAT validation | TCTTTCCTCATTGTGTGTCTTGAAG |  |
| ESP192 | ypk2 rv NAT validation | GTTCTACTTGAGTTCGCAACACG |  |
| ESP225 | slt2 fwd replacement template | GAAACATCAGTGGCTGGACGGG |  |
| ESP226 | slt2 rv replacement template | TCGAAATGAGTCAAAGCTGGCC |  |
| ESP227 | slt2 fwd NAT validation | CGTAGTGATGCCTAGATGCCC |  |
| ESP228 | slt2 rv NAT validation | GCATCTCCTCATCAGTCAGGTAG |  |
| ESP185 | slg1 fwd replacement template | AGAAAACGCGAAAAGGAGAGAG |  |
| ESP186 | slg1 rv replacement template | TGCAGCAAAGCCAGATATTAGG |  |
| ESP187 | slg1 fwd NAT validation | CACTACCACTCACACGTCACG |  |
| ESP188 | slg1 rv NAT validation | ATGCGGATATTGTTCTGGGTATTG |  |
| ES9B | NAT rv internal primer | GTATTCTGGGCCTCCATGTC |  |
| ES10B | NAT fwd internal primer | GTGAATGCTGGTCGCTATAC |  |
| FKS1F | fks1 fwd primer | ACGTCGCTTCTCAAACCTTC | This  study |
| FKS1R | fks1 rv primer | CACCACCAACAGTCAAATCG |  |
| RGFKS2F | fks2 fwd primer | ATGCTGTAGCAGCTGTGAC |  |
| RGFKS2R | fks2 rv primer | TAACGAGCACCACCCACAG |  |
| ITS1 | rDNA species identification fwd | TCCGTAGGTGAACCTGCGG | (21) |
| ITS4 | rDNA species identification rv | TCCTCCGCTTATTGATATGC |  |
